# Report of the Proceedings of the Medical Association of Georgia, for 1860

**Published:** 1860-05

**Authors:** A. G. Thomas

**Affiliations:** Sec. M. A. of Ga.


Medical and Surgical Journal.

Vol. V.] MAY, 1860. [No. 9

### ORIGINAL COMMUNICATIONS.

### ARTICLE I.

*Report of the Proceedings of the Medical Association of Georgia, for* 1860.

The Medical Association of Georgia, assembled in the City Hall at Rome, at eleven o'clock, April 11th, 1860.

The House being called to order by the President, Dr. F. S. Colley, the deliberations of the body were introduced with prayer, by Rev. Mr. Jones, of Rome.

The roll being called, the following regular members answered to their names:

J. G. Westmoreland, J. P. Logan, J. N. Simmons, Hayden Coe, W. F. Westmoreland, A. G. Thomas, H. W. Brown, of Atlanta; Eben Hillyer, R. C. Word, T. J. Word, of Rome; DeSaussure Ford, Robert Southgate, of Augusta; A. M. Boyd, W. A. Culbertson, of Cave Spring; F. S. Colley, of Monroe; J. N. Coe, Flat Rock; S. W. Burney, of Forsyth; J. T. Banks, of Griffin; J. R. McAfee, of Dalton; W. C. Brandon, of McGuire's Store; W. P. Bond, of Lithonia, and Johnson Matthews of Yellow River.

The minutes of the last Meeting were then read and confirmed. Rules being, on motion, suspended, the following candidates were, on written application, accompanied with suitable vouchers, elected to membership in this Association: J. B. Underwood, John M. Gregory, A. B. Gregory, Wm. Terrell, J. King, J. W. B. Nowlin, J. C. Reese, T. F. Jones, of Rome; L. Price, D. F. Forman, —— Wall, Z. L. Waters, of Calhoun; B. B. Brown, J. A. Black, of Dalton; M. F. Crumley, Atlanta; V. M. Hodgson, Villa Rica; E. A. Ware, Floyd county; D. R. Richardson, Monroe, Walton co.; N. C. Mason, Cass Co.; E. L. Connally, Fulton Co.; G. M. McDowell, Pike Co.; J. N. Smith, Tilton; A. H. Shi, Newton Co.; M. R. Ballenger, Floyd Springs.

Election of officers being in order, the President ordered a ballot for President. Dr. Burney proposed the name of Dr. H. Coe, of Atlanta. Dr. T. J. Word proposed the name of Dr. Robt, Southgate of Augusta. Dr. Southgate's name being withdrawn, Dr. H. Coe was, on ballot, declared unanimously elected President for the current year. Dr. Logan proposed the name of Dr. T. J. Word, of Rome, for first Vice President. On ballot, Dr. Word was declared unanimously elected 1st V. P. Dr. Hillyer proposed the name of Dr. Robt. Southgate, of Augusta, for second Vice President.—On ballot, Dr. Southgate was declared unanimously elected 2d V. P. Dr. Banks proposed the name of Dr. A. G. Thomas, of Atlanta, for Cor. and Rec. Secretary and Treasurer.—On ballot, Dr. Thomas was declared unanimously elected.—Dr. Logan proposed the name of Dr. S. W. Burney, of Forsyth, for Orator at the next meeting. On ballot, Dr. Burney was found to be unanimously elected next Orator. On ballot for Alternate Orator, Dr. W. F. Westmoreland was declared unanimously elected.

On motion, the President appointed as a Committee, to induct the President elect into the Chair, Drs. Banks, Hillyer and A. M. Boyd.

Dr. Colley, on retiring from the Presidency, delivered a short, but beautiful and appropriate address—very strikingly marking some of the causes which tend to the lowering of the standard of the Medical Profession.

Dr. Coe was then inducted into the Chair, and assumed the duties of the office, by making a few chaste and happy remarks.

On motion, the Association adjourned till 3 o'clock, P. M.

---

AFTERNOON SESSION, 3 o'clock, P. M.

House was called to order by the President. The President called for the introduction of business requiring early action. Dr. Logan moved that, the Association appoint 7½ o'clock, P. M., as the hour for the delivery of the Annual Oration—carried. Dr. Boyd moved that, the President appoint a Committee of five to nominate Delegates to the American Medical Association—motion carried. Committee, Drs. Colley, Logan, Burney, Southgate and Banks.

On motion of Dr. Colley, Association determined to send two Delegates to the Convention for revision of Pharmacopœia, to assemble in Washington, D. C., in May next. Dr. Logan proposed the names of Prof. I. P. Garvin, of Georgia Medical College, and Prof. J. G. Westmoreland, of Atlanta Medical College. On ballot, Drs. Garvin and Westmoreland were unanimously elected Delegates.

Dr. Burney offered the following resolution :

*Resolved* That, Dr. Colley be requested by this body, to furnish a copy of his address delivered this day, on retiring from the Presidency, to some one of the Medical Journals of the State, for publication.

Resolution unanimously adopted.

Reports of Auxiliary Societies being called for, no report was offered. Rules being suspended, Dr. J. G. Westmoreland moved that, the Committee on Nomination of Delegates to American Medical Association, be instructed to select eighteen names. Dr. Banks offered as a substitute, that as many be selected as this body is entitled to. Substitute being put to vote, was carried.

Order being resumed, Written Communications were called for—no report.

On motion of the Secretary, Voluntary Written Communications were called for.

Dr. Boyd presented a report of a case of Ulceration of Cervix Uteri.

Dr. Southgate then presented a very interesting Essay, entitled, The Tendency to Abandon the Practice of General Blood-letting in the Treatment of Disease—is it evidence of an advance or retrograde movement in Therapeutics?

On motion, Association adjourned till 9 o'clock, April 12th.

At 7½, P. M., Association listened to the very elegant Annual Address by Dr. H. W. De Ford, of Augusta.

---

SECOND DAY, 9 o'clock, A. M., April 12th.

Minutes of yesterday were read and confirmed.

Regular order being taken up, Dr. Burney presented a case of Laceration of Uterus.

Dr. T. J. Word moved that, the authors of the papers presented to this meeting, be requested to furnish them for publication in some of the Medical Journals of the State—carried. Dr. R. C. Word offered the following resolution:

*Resolved*, That members of this Association, having unfinished Essays designed for this occasion, shall be allowed to complete them and have them published by order of this body.

Oral communications being called for, W. F. Westmoreland, M. D., Prof. of Surgery in Atlanta Medical College, presented an instrument, of his own invention, for making a section of small strictures of the urethra without dilatation, with bad case successfully treated.

Prof. W. F. Westmoreland presented a report of various experiments upon animals, no brief synopsis of which is here introduced, with a view to determine the practicability of ligating arteries with silver wire, and the closure of wounds of the external surface and internal organs, as intestines, &c., with the silver suture.

Experiment 1.—Subject, a pup five or six months old.—Left carotid artery ligated. Result—Death of the pup from severing the artery by scratching. Exp. 2.—Pup, six weeks old; right femoral artery ligated. Result successful, ligature encysted. Exp. 3.—Dog, two or three years old; right femoral artery ligated. Result successful, artery obliterated; ligature beautifully encysted. Exp. 4.—Dog, two or three years old; Longitudinal incision in small intestine, two and a half inches in length. Result—Dog found dead in forty hours after operation, bowels protruding through external opening, wound united. Exp. 5.—Dog, very old; right femoral artery ligated. Result successful, artery obliterated, ligature encysted. Exp. 6.—Dog, two or three years old; incision one and a half inches long in intestine. Result successful, wound closed, suture encysted. Exp. 7.—Dog, two years old; right common iliac artery ligated. Result—Artery obliterated, ligature encysted. Exp. 8.—Spaniel, two or three years old; longitudinal incision, two inches, made in small intestine. Result—Bowel united, wire encysted. Exp. 9.—Dog, two years old; attempt to ligate abdominal aorta. Result—Rupture of aorta ligated, lived only a few hours. Exp. 10.—Spaniel, young; abdominal aorta ligated. Result successful; aorta obliterated, ligature encysted. Exp. 11.—Bitch, supposed five or six years old; section of small intestines, one and a half inches, removed. Result—Lived forty hours, wound united.

In some of these cases, Chloroform was administered—in the others, Sulphuric Ether.

Dr. Dugas, through Dr. De Sausure Ford, presented a report of a case of Epesioraphy, and also a report of a case of Staphyloraphy. Dr. Colley reported "an anomalous case of delivery." Dr. W. F. Westmoreland reported success in restoring respiration in animals, when chloroformization had been carried too far, by artificial respiration, effected by the introduction into the trachea of a large elastic bougie through which air was forced by a common hand bellows.

Report of Committees appointed at last meeting to report to this meeting, in order.

Report of Committee on revision of Constitution and By-Laws, called for. No report.

Report of Committee on Medical Literature, called for.—No report.

On motion, both these Committees were continued, with instructions to report at next meeting.

Report of Committee on nomination of delegates to American Medical Association, called for. Committee reported the following names: Drs. Southgate, T. B. Phinizy, L. D. Ford, H. H. Steiner, Augusta; Drs. J. P. Logan, H. Coe, J. N. Simmons, Atlanta; Drs. J. T. Banks, E. F. Knott, T, M. Darnall, Griffin; Drs. W. G. Bulloch, Juriah Harris, Savannah; Drs. Robt. Battey, T. J. Word, Rome; Drs. F. S. Colley, D. R. Richardson, Monroe; Dr. S. W. Burney, Forsyth; Dr. W. W. Flewellyn, Columbus; Dr. R. A. T. Ridley, LaGrange; Dr. J. R. McAfee, Dalton; Dr. G. L. McClesky, Athens; Dr. W. S. Meire, Madison; Dr. E. O. Ware, Cartersville; Dr. Alex. Means, Oxford; Dr. S. P. Lumpkin, Watkinsville.

On motion of Dr. Boyd, report was received and adopted.

Selection of place for next meeting being in order, Dr. J. N. Coe, proposed Atlanta; Dr. T. J. Word proposed Macon. Dr. J. G. Westmoreland moved that a Committee be appointed to report a place for next meeting. Dr. Hillyer moved, as a substitute, that a vote on that question be taken by ballot. Motion carried.

On ballot, Atlanta was declared the place chosen for holding next meeting of this Association.

On motion of Dr. Boyd, the Chair appointed a Committee of three to nominate Essayists for next meeting. Committee —Drs. Boyd, Logan, and Southgate.

Dr. T. J. Word offered the following resolution:

*Resolved*, That the experiments of Prof. W. F. Westmoreland are highly commendable, and it is hoped they will be continued, and result in great good to the Profession.

Dr. T. J. Word moved that sections 3rd and 4th, Art. 1st, Chap. 2, of the code of Medical Ethics, be published with the minutes of this meeting. Carried.

Dr. W. F. Westmoreland moved that the Secretary be instructed to have 500 copies of the Constitution and By-Laws of this organization, published, and distribute them to its members.

Dr. Logan moved, as a substitute, that the Committee on Constitution and By-Laws be empowered to revise the same, and publish 500 copies.

Dr. Brown moved to amend, by adding that the Secretary be required to distribute the copies so published to members.

Dr. Colley moved to table the motion. Dr. Colley's motion lost.

The original motion, as amended, was then put to vote, when the vote resulted in a tie. The President voting in favor of the motion, it was carried.

On motion, the Treasurer was ordered to draw on Treasury for funds to be furnished to Committee on Constitution and By-Laws, to pay for 500 copies, to be published as soon as practicable.

Dr. R. C. Word offered the following Resolution:

*Resolved*, That notice be now given that at our next meeting a vote shall be taken to determine a place for the permanent location of this Association.

Dr. Ford offered as a substitute:

*Resolved*, That notice be now given that at our next meeting, this Association determine whether or not this Association shall be permanently located, and if decided to locate, the place shall be chosen.

The substitute being put to vote, was carried.

The Committee on Essayists reported the following list of names:

King, Battey, Hillyer, T. J. Word, Rome; W. F. Westmoreland, D. C. O'Keefe, Atlanta; Doughty, Augusta; B. B. Brown, Dalton; J. T. Banks, Griffin.

On motion, report was received and adopted.

Dr. Logan moved that the Committee on Constitution be required to revise the list of names of members. Carried.

On motion of the Secretary, that part of the proceedings continuing the Committee on Constitution, was reconsidered.

On motion of the Secretary, the President was required to appoint a new Committee of five to revise Constitution and By-Laws.

Dr. Ford moved that the Committee be appointed from one place. Motion lost.

The President appointed as that Committee, Drs. A. G. Thomas, H. W. Brown, L. D. Ford, H. F. Campbell, J. T. Banks.

On motion of Dr. Boyd, the President appointed as the Committee of Arrangements for next meeting, Drs. H. W. Brown, J. G. Westmoreland, J. F. Alexander, D. C. O'Keefe, J. N. Simmons.

Dr. W. F. Westmoreland moved that a Committee on Finance be appointed to devise some means of raising funds for the use of this Association, and to report at next meeting; carried. Committee—W. F. Westmoreland, F. S. Colley, R. C. Word.

Dr. R. C. Word offered the following Resolution:

*Resolved*, That the President appoint a Committee of five to memorialize the Legislature of Georgia, at its next session, to abolish the professional Tax upon Physicians, and to urge the passage of an act requiring the Inferior Court of each County to set apart such portion of the County Tax as the Grand Jury shall recommend, to purchase drugs for the benefit of the poor. Adopted.

Committee—Drs. R. C. Word, B. B. Brown, J. G. Westmoreland, Burney, Southgate.

Dr. Simmons offered the following Resolution:

*Resolved*, That the thanks of this Association be tendered to the city authorities of Rome, for the use of their Hall, and to the Physicians and citizens of the city, for their kind attention to the members of this body during its present session.

Resolution adopted.

On motion, the Association adjourned to meet in the city of Atlanta, on the second Wednesday in April, 1861.

---

*From the Code of Medical Ethics, Chapter Second.*

Section 3.—It is derogatory to the dignity of the Profession, to resort to public advertisements, or private cards, or handbills, inviting the attention of individuals affected with particular diseases—publicly offering advice and medicine to the poor, gratis; or promising radical cures; or to publish cases and operations in the daily prints, or to suffer such publications to be made; to invite laymen to be present at operations—to boast of cures and remedies—to adduce certificates of skill and success, or to perform any other similar acts. These are the ordinary practices of empirics, and are highly reprehensible in a regular physician.

SECTION 4.—Equally derogatory to professional character, is it, for a physician to hold a patent for any surgical instrument or medicine; or to dispense a secret *nostrum*, whether it be the composition or exclusive property of himself or of others. For if such nostrum be of real efficacy, any concealment regarding it is inconsistent with beneficence and professional liberality; and if mystery alone can give it value and importance, such craft either implies disgraceful ignorance or fraudulent avarice. It is also reprehensible for physicians to give certificates attesting the efficacy of patent or secret medicines, or in any way to promote the use of them. A. G. THOMAS, Sec. M. A. of Ga.

